# Education as a Social Determinant of Health: Issues Facing Indigenous and Visible Minority Students in Postsecondary Education in Western Canada

**DOI:** 10.3390/ijerph10093908

**Published:** 2013-08-28

**Authors:** Janki Shankar, Eugene Ip, Ernest Khalema, Jennifer Couture, Shawn Tan, Rosslynn T. Zulla, Gavin Lam

**Affiliations:** 1 Faculty of Social Work, University of Calgary, 444 11044, 82 Avenue, Edmonton, AB T6G0T2, Canada; E-Mails: stan@ucalgary.ca (S.T.); gavincares@gmail.com (G.L.); 2 Department of Community Studies, Faculty of Health and Community Studies, NorQuest College, Edmonton, AB T5J 1L6, Canada; E-Mail: eugene.ip@norquest.ca; 3 School of Public Health, University of Alberta, Edmonton, AB T6G 1C9, Canada; E-Mails: ekhalema@ualberta.ca (E.K.); rzulla@ualberta.ca (R.T.Z.); 4 Human Sciences Research Council (HSRC), Private Bag X41, Pretoria 0001, South Africa; 5 Department of Built Environment, Population and Development Studies, University of Kwazulu-Natal, Durban, KZN, 4001, South Africa; 6 Department of Sociology, University of Ottawa, Ottawa, ON K1N 6N5, Canada; E-Mail: jennifer.couture@uottawa.ca

**Keywords:** low income minority youth, postsecondary education, social determinants of health

## Abstract

The level of educational attainment is increasingly being recognized as an important social determinant of health. While higher educational attainment can play a significant role in shaping employment opportunities, it can also increase the capacity for better decision making regarding one’s health, and provide scope for increasing social and personal resources that are vital for physical and mental health. In today’s highly globalized knowledge based society postsecondary education (PSE) is fast becoming a minimum requirement for securing employment that can afford young adults the economic, social and personal resources needed for better health. Canada ranks high among OECD countries in terms of advanced education, with 66% of Canadians having completed some form of postsecondary education. Yet youth from low income indigenous and visible minority (LIIVM) backgrounds continue to be poorly represented at PSE levels. The current study aimed to understand the reasons for this poor representation by examining the experiences of LIIVM students enrolled in a postsecondary program. Findings show that the challenges they faced during the course of their study had an adverse impact on their health and that improving representation of these students in PSE will require changes at many levels.

## 1. Introduction

The World Health Organization’s Commission on the Social Determinants of Health has presented overwhelming evidence that health and quality of life are socially determined and that entrenched health inequities among people originate not so much from lack of hospital or community based services as from the failure of Governments to address the “social determinants of health” [1]. These refer to the economic and social conditions that shape the health of individuals, communities, and jurisdictions [2]. While health care services, food, housing and a social safety net are important social determinants of health, recent reports show that the level of educational attainment is a strong predictor of long term health and quality of life [3].

There are several interrelated pathways through which educational attainment is linked with health [4]. Higher educational attainment can lead to improved health as more educated individuals will most likely make better-informed, health related decisions for themselves and their families [5,6]. Higher educational attainment can also play an important role in health by shaping employment opportunities, which are the major determinants of economic resources. More educated individuals will experience lower rates of unemployment, which is strongly associated with worse health and higher mortality [7]. Higher educational attainment can also affect health by influencing social and psychological factors like greater perceived personal control [8], which has frequently been linked with better health and health-related behaviors [9,10], higher relative social standing and increased social support [11]. All these factors are associated with better physical and mental health [12].

In today’s highly globalized knowledge based society postsecondary education (PSE) is fast becoming a minimum requirement for securing employment that can afford young adults the economic, social and personal resources needed for better health and quality of life. Young adults who do not pursue postsecondary education are likely to experience a lower socio economic status (SES) than those who acquire further education and skills [13]. In 2005, 66.9% of Canadians with a PSE qualification reported being in excellent or very good health *vs.* 42.9% of those without a high-school diploma. In 2006, the Organization for Economic Co-operation and Development (OECD) reported that the percentage of people claiming positive life satisfaction increased with educational attainment [3]. Advanced education is also associated with positive societal outcomes including higher productivity, innovation, economic growth and stronger communities [14].

Canada ranks high among OECD countries in terms of advanced education with 66% of Canadians aged 25 to 64 having completed some form of post-secondary education. However, youth from indigenous and low income backgrounds are under-represented at postsecondary levels [3]. Increasingly immigrants, especially from some “visible minority” backgrounds are joining the ranks of low income earners increasing their risk for poor health and quality of life. The Employment Equity Act defines “visible minorities” as “persons, other than Aboriginal peoples, who are non-Caucasian in race or non-white in color.” According to this Act visible minorities include Chinese, South Asian, Black, Filipino, Latin American, Arab, West Asian, Japanese and Korean [15]. Low income among visible minority immigrants, reached decade-high levels in 2002 and 2003, about 3.5 times higher than the rate among the Canadian-born population, despite the fact that 52% of those in chronic low income were skilled economic immigrants, and 41 percent had university degrees [16].

Given the unequivocal benefits of PSE for health and well-being, and the number of students from low income backgrounds who are unable to access PSE, the experiences of those from these backgrounds who make it to postsecondary educational institutions becomes very important. The focus of this study is on the school experiences of students from low income indigenous and visible minority (LIIVM) backgrounds enrolled in a postsecondary educational setting in Western Canada and the influence of these on their health and well-being.

## 2. Literature Review—Barriers to PSE for Students from Low Income Backgrounds

Students from low income (LI) backgrounds face several inter related barriers to pursuing and persisting with postsecondary education. *Lack of financial capital* is one of the most widely cited barriers. A steep increase in undergraduate student fees in the last fifteen years has lured many youth from low income backgrounds to join the labor force instead of undertaking postsecondary education [17]. *Lack of proximity* to postsecondary institutions, which are usually located in major cities and urban areas, and *transport problems* are other factors that can substantially impact the costs associated with obtaining this education, especially for those low income students living in rural and remote areas. *Parental levels of educational attainment* play an important role in youth entering and persisting with postsecondary education. Studies show that children of parents who have completed postsecondary education are substantially more likely to enroll in higher education than those whose parents’ educational attainment did not exceed high school [18]. This particularly disadvantages low income indigenous youth as most of their parents and grandparents were part of the residential school system and could not access higher education [19,20].

### Additional Barriers Faced by Students from LIIVM Backgrounds

While a significant number of students from low income backgrounds experience difficulties accessing postsecondary education, those from LIIVM backgrounds may face additional barriers arising from factors like systemic discriminatory practices and acculturative stress that can be experienced much more intensely by immigrants from visible minority backgrounds [21,22]. These can have an adverse impact on psychological health and well-being. Students from some visible minority groups entering postsecondary study may be racialized as deviant, delinquent, gang members or lacking in academic potential [23,24]. Such stereotyping can result in students from these groups experiencing negative counseling from teachers and counselors. They may receive little encouragement from departmental heads to apply for resources like scholarships because of preconceived assumptions that they will be incapable of handling the demands of higher study despite getting good grades. Such experiences can dampen their career aspirations and contribute to low academic self-confidence.

For many indigenous and visible minority students the mainstream University may represent an impersonal, intimidating and hostile environment in which little of the cultural knowledge, traditions and values they bring are recognized or valued. Kirkness and Barnhart [25] highlight that “indigenous students who enter these institutions are expected to leave behind the cultural assumptions of their world, develop a new consciousness and orientation and assume the trappings of a reality very different from their own. They are expected to adapt to the long established conventional practices, norms, and policies designed to serve the mainstream Anglo culture and perform at levels comparable to mainstream students. Those who fail to adapt or perform are defined by the University in terms such as low achievement, high attrition and poor retention, thus placing the onus of accommodation on the student rather than on the University’s policies, and organizational structure”. The same may apply to students from low income visible minority backgrounds.

Despite these barriers an increasing number of students from LIIVM backgrounds are enrolling in postsecondary education to improve their chances of gaining better jobs and wages. This includes a significant number of mature aged (25 years and above) learners. Many of these students are attracted by state-sponsored financial assistance schemes that are offered by liberal Governments to mitigate some of the educational barriers faced by students from low income backgrounds [14]. Many LIIVM mature aged students often have family responsibilities, are caregivers and some may be single parents [26]. The responsibilities that come with single parenthood and/or familial responsibilities can often impact their financial circumstances, study capacity and physical and psychological health. Canadian research that examines the postsecondary school experiences, circumstances and world views of these students is scarce [27,28]. Given that postsecondary education is fast becoming a minimum requirement for securing employment, an understanding of the financial, social, physical and psychological challenges these students face as they navigate their way through the demands of study and the study environments can help in identifying the kinds of supports they will need to optimize their potential to achieve their educational goals.

The purpose of the current study was to examine the aspirations of LIIVM enrolled in postsecondary study, the challenges they faced during the course of their study program and their perspectives on how these influenced their health and well-being. Research on minority students has mostly been conducted at postsecondary institutions that serve predominantly mainstream students. For the purpose of this study a community college that serves a large population of low income students from diverse cultural backgrounds was selected. The following specific questions were examined: (a) what are the aspirations of indigenous and visible minority students enrolled in a postsecondary educational program? (b) What challenges did indigenous and visible minority students face during the course of their study? (c) What are the students’ perspectives on how these challenges influenced their health and well-being?

## 3. Method

A qualitative methodology informed by interpretive phenomenology [29] was employed to examine the aspirations, challenges and health concerns faced by 14 students from indigenous and visible minority backgrounds. The interpretive phenomenological approach permits the use of theoretical perspectives when these will not in any way have a biasing effect on the participants’ narratives [30]. In this study we drew from Critical theory (CT) in helping us to interpret some of the findings. CT in the context of education seeks to analyze how oppressive structures, institutional arrangements and governance may impact students, especially those from marginalized backgrounds and serve as barriers to educational attainment [31].

The study was open to all students from indigenous and visible minority backgrounds attending a Human Services program in the community college. Students were recruited with the assistance of the program coordinator who disseminated information about the study to students in their classrooms. Study information was also displayed on the school notice boards. Students who expressed interest to participate in the study provided their contact details to the program coordinator who in turn provided these to the research investigators. A total of 14 students—seven indigenous and seven immigrant students—volunteered to participate in the study. This included 11 female and three male students.

In-depth semi-structured face to face interviews were conducted by the first author and two graduate research assistants trained in qualitative research interviewing. Each interview lasted for a maximum of 2 h. Interviews started with broad open ended questions: “Tell me what a typical day at school is like”; “Tell me why you decided to go back to school; “Tell me about some challenges you face as a student (financial, relationship with peers, teachers, counselors, administrative staff, student advisors; financial assistance, taking care of your children, other relatives); “Can you share with me how some of these challenges make you feel or affect your health; “What supports do you have”? All interviews were audiotaped after receiving consent and transcribed verbatim.

Data were analyzed using thematic analysis. Each transcript was read several times independently by two members from the research team in order to obtain an overall understanding about participant experiences and challenges and how they coped with these. In keeping with qualitative data analysis the transcripts were coded into meaningful segments and then combined into categories to generate themes [32]. The coders were able to achieve a 90% agreement on the themes. Measures taken by the researchers to increase the reliability and validity of the qualitative data included maintaining an audit trail for the whole research process, member checking after the interviews, keeping field notes, interviewing until saturation of data was reached and including several direct quotes while discussing the results of the study.

Except for three, all students in the study sample received student financial assistance from the Provincial Government. Although the study did not specifically target mature aged or first generation migrant students, all those who agreed to participate were mature aged (their age ranged from 25–52 years) and all the immigrant students were first generation Canadians who had migrated within the last 10 years. The majority of the students (11/14) had caregiving responsibilities—they had young children or had caregiving responsibilities for extended family members. Most of the study participants (11/14) worked between 5 to 8 h a day to supplement their earnings.

## 4. Results

We present below the themes in response to each of the research questions.

### 4.1. The Aspirations of Students Enrolled in Post-Secondary Education

#### 4.1.1. Hope for a Better Future

Students aspired to get jobs as welfare and community workers at the end of the program. All students were in agreement that the program they were in would help them to get jobs that could improve the quality and standard of life for their family members. Some students said that the program of study provided the opportunity to move out of dead end menial jobs that offered little scope for economic and social advancement. In the case of some others it offered a way out of Government social assistance which they perceived as oppressive and disempowering. As commented by one student:

“*I was tired of being on social assistance because it’s like they have to know your life. They dictate your life*, *right*, *depending’ on which worker you have*, *right. They ask questions like about income*, *about umm who’s all living’ with you and stuff like that. And some of it’s personal you know*, *and some of them they don’t need to know….*”

#### 4.1.2. Giving Back to Their Community

Many immigrant student participants had experienced difficulties in the process of settling in Canada. Some had come in as refugees from war torn countries and had experienced difficulties navigating their way through services and accessing help from service providers. These students expressed that gaining employment as community workers would fulfill their desire to help new immigrants and refugees. This is captured well in the following excerpt:

“*I come across a lot of the Africans that are coming in and they express a lot of problems with integration into the mainstream and there is the need for somebody that they will be comfortable with*, *their own—they say that they feel intimidated over the time you know and that somebody of their own would be very comfortable*.”

#### 4.1.3. Upgrading Qualifications

Three immigrant students already had graduate degrees from overseas that were not recognized in Canada. These students had enrolled in the program to upgrade their qualifications and gain employment that was more in line with their interest and previous training. One of these students was a qualified teacher with several years of teaching experience overseas. Another student who had technical qualifications had worked at odd jobs for 11 years after migrating to Canada in order to support his family. In the case of some students their caregiving responsibilities had delayed their entry into postsecondary study and they were looking forward to a new break in their lives.

#### 4.1.4. Being Good Role Models

An additional reason mentioned by some students for entering postsecondary study was the desire to be good role models for their growing children. This is illustrated by the following excerpt:

“*I wanted to show my daughter who’s gonna be ten this year that she could go to school*, *you know*…* that she doesn’t have to work at low-paying jobs or*, *or be on welfare*, *right… I wanted to change all that for her.*”

Thus, for many students attaining postsecondary education meant breaking out of the lived experience of social subordination from an intergenerational perspective. The above excerpt also suggests that while children may inhibit many women from poor socioeconomic backgrounds from undertaking and persisting with study [33], children can be a strong incentive to pursue study and have a positive impact on the parent’s education goals [34]. In summary students had taken up postsecondary study for a number of reasons ranging from wanting to improve their career prospects and financial circumstances and afford a better standard and quality of life for their family, starting a new phase in their lives, giving back to their community, and wanting to be good role models for their children.

### 4.2. Challenges Faced during the Course of Their Study Program

#### 4.2.1. An Oppressive Financial Assistance Scheme

As stated earlier, the majority of students in the program (11/14) were receiving state sponsored financial assistance to pursue postsecondary study. An ironic theme that emerged from the research findings is how a programmatic resource presumably designed to target financial barriers to education turned out to be a significant health determinant. Three concerning realities emerged from narratives informing this theme: first, inadequacy of assistance, second, conditions of the financial assistance scheme and third, information control. Working together, these lived realities had significant health implications for the students.

Inadequacy of assistance is about the student’s everyday predicament that the financial assistance scheme falls short of supporting their needs arising from the multiple realities of their lives. A student summarized this in a way that speaks to voices from other peers in this research study:

“*If the intent of the assistance is to help people like me not become burden on the system but to become a productive member they were going to give me something that will sustain me to go through the program. At the end of the day after the tuition fee*, *I was left with $840 to pay for my rent*, *transport and lodge. Most students in my course were breaking down…*”

The participants in this study were matured adults trying to empower themselves through education; this personal campaign was typically waged notwithstanding a background of complex, troubling life histories that continue to bear on them with hefty psychological baggage and pressing everyday material responsibilities. The indigenous student participants represented those who had grown up in impoverished reserve households where schooling has been inter-generationally an endeavor of self-actualization far beyond everyday immediate material deficits and behavioral crises that preoccupy life on reserves. Others represented a struggling immigrant life following years of harsh existence as in cases of running away from oppressive regimes and of eking out a horrific existence in refugee camps. With being students, current life-styles for many of these students encompassed intercepting realities such as single parenthood, spousal violence, homelessness, poverty trying to stay clean from an addiction. All these past and current lived realities add up to a typical student life, as reported by study participants.

By all accounts as represented by the above quote, the financial assistance scheme—however, it awards according to individual needs—made little room if any at all to accommodate unplanned situations and unexpected monetary demands that imaginably pop up in the everyday lives of students mired in their respective internal and external psycho-social environments that often lend limited order and predictability. The students’ narratives highlighted situations such as taking refuge from family violence, lending money to a family member in crisis, an unplanned move of residence, expenses associated with moving and securing rental of another apartment, expending on children’s extra-curricular recreation and interference from child welfare services for having left their children alone at home after school. When situations such as these arise the monthly budget associated with the financial assistance scheme gets subverted and these students are more at risk of confronting and coping with a chaos of stressful concerns and physical exertion. In order to deal with these the majority of the students on assistance were forced to take up full or part time employment to top up the financial assistance and this created much physical and mental strain.

Additionally the conditions of receiving financial assistance for schooling required the students in this study to enroll in full-time studies and to pass all five courses they had to enroll in each semester. Perfect class attendance is expected with little room for excusing absence. While the financial assistance scheme is invariably advertised as a support to these students, the agenda of behavioral management slips into the rule book for assistance applicants and recipients. The following conditions of a typical financial assistance scheme illustrate such an agenda [35].

Career investigation: Qualified students must have a set of employment goals…

Referral to training: Student advisor to approve your training plan…

Commencement of training: Training commences and you enter Service Management. This is your support system. It monitors your attendance and progress while you are in school.

Completion of training: you…start looking for work

Follow-up: you will be contacted twice over a six-month period, once at three months and once at six months, for a report on your job search or employment situation

In other words, applying to and receiving from a financial assistance scheme is allowing oneself to be constructed into a client of the scheme where one enters into a contract to complete a job according to external expectations and criteria. In being “clientized”, the student agrees to be assessed, decided for, monitored and interestingly, shepherded and observed beyond the schooling period, which the scheme only funds and therefore should limit itself to be concerned about.

In this analytical light, being a scheme client comes with additional workload related to rules and expectations connected to state-sponsored help. Based on what the students in this study described, the daily regime they made effort to maintain to meet the schematic conditions ironically often competed for attention with heavy school work and from time to time, unforeseen, unplanned life events or crises and financial issues within their lives of multiple realities. These conditions became overarching stressors piled on top of other ones—on-going and potential.

A third lived reality about the state-sponsored financial assistance scheme is students’ experience that funding bureaucrats at their college were less than helpful in offering information that lends options to arising circumstances that could jeopardize their continuation in school. By the students’ accounts, “if you don’t ask, I don’t tell” guideline seemed to be a part of the operational model of these bureaucratic staff who, ironically, the students commonly referred to as their “advisors”.

A student who was taking a full load of five courses as required by the financial assistance scheme and still had to take a 5 h nightly work shift to supplement his income to support dependent children experienced a financial crisis toward the final part of the program. He ended up quitting before completing the studies. By other students’ accounts, such financial schemes typically have provisions to help recipients to get over financial humps in times of extraordinary needs. However, as one female student shared her experience from approaching one of her college’s fund administrators, “I was made to feel as if they’re saying to me “we are doing you a service by giving you assistance”. She never ended up getting the help sought for her financial difficulties. In the end, she did her own on-line research and became aware of provisions within the financial scheme that the administrator could have explored with her without making her feel like society’s burden. In her own words,

“*I had to dig in*, *even though the system is there*, *you have to know the vocabulary to use because it’s controlled to a point where they don’t tell you until you ask*.”

##### Implications of Financial Assistance Schemes on Student Health and Well-Being—A Critical Theory Perspective

The linkage of the three lived realities arising from student participation in the financial assistance scheme and the expectation that students must pass all the courses in order to maintain eligibility, caused considerable physical and mental stress that often affected their school performance. This was very well articulated by one student who had observed her peers struggle with (in her words) “mental focus” in the classroom. She further explained: “half the time in class they worry about their lives, their jobs, their kids”. From a critical theory perspective, the linkage of the three lived realities arising from student participation in the financial assistance scheme to their health is “extra-local”—to borrow an analytical descriptor from Dorothy Smith’s sociology based on institutional ethnography [36]. Applied here, extra-locality refers to how the organization of the scheme structures the experience of adverse health impact for the student-clients outside the discourses in addressing any recipients’ financial issues.

Yet, the physical and mental toll related to staying afloat in the sea of pressure to abide by rules and expectations of the scheme are everyday lived human conditions. Extra-locality of health impact by being student-clients of the scheme therefore becomes a ruling device whereby students’ physical and mental strain and stress from managing themselves as clients are by necessity rendered unrelated, irrelevant and therefore, invisible within the social relations of the scheme involving the administrators/advisors and student-clients. In this sense, all the strain and stress that go into abiding by rules and meeting expectations become extra-localized as students’ personal responsibilities to manage. This further allows a discourse of personal life-style management to be dished out to funded students, which would suggest that self-care and staying healthy is part of the person’s duty so that s/he is able to have full attendance, to pass courses, to complete program, to start looking for work and to stay employed. In other words, the linkage to health is now effectively shifted to being with the funded student’s integrity as clients and away from the scheme.

Torjman in her 2009 report *Student Aid Meets Social Assistance* invokes the term “supplementarity” to explain that student financial assistance schemes typically go by the principle that they only act as a supplement to available resources [37]. In other words students who access these schemes are expected to work and use their savings or seek family contributions to help with their educational costs. In this sense, supplementarity effectively reinforces the neo-liberal expectation that student-clients must only be assisted in a manner they have to work for what they are provided by the welfare institution. Significant here for an analytical understanding is that this expectation is grounded in the idea that rather than the system within which the individual is made to assimilate, it is the individual him/herself that has to assume full accountability for own success and failure. That is success and failure in all aspects of life, including health. Thus, by neo-liberal tenets, the system hardly features as a factor of health of individuals who are subjected to the system’s functioning.

In this ideological light, sense is readily made of the financial assistance scheme that it has a mission to (through implementation of a regime of rules, conditions and expectations) route student-clients to gainful employment; hence the steps cited above in terms of what the scheme in question is expected to do with and to the student-clients. The irony of it all is that as a helping scheme with a public face for the idea of education being a determinant of health, the scheme in practice contributes to constructing oppressive conditions that subvert student-clients’ health and well-being and therefore their effort for academic success. If education is a social determinant of health, financial assistance schemes such as these may become counter-productive as evident by the findings in this study and students are put at risk of having health issues by virtue of being clients of a “helping” scheme.

#### 4.2.2. The Culture of Teaching and Learning

Almost all students shared that the program, which was linked with the financial assistance scheme lacked the flexibility to accommodate their life circumstances. Additionally students expressed their discontent about the teaching and assessment practices that were largely based on “reading texts and writing assignments”. Often students had to hand in 3 to 4 essays in a week and this called for several hours of reading from different texts. This meant working through the night which left them constantly tired the following day. One student disclosed that the emphasis on essays forced some students to purchase online essays and submit them as their own. Some students expressed that teaching and assessment practices were very much modeled on western ways of learning. Their views are well captured in the following comment from one student:

“*Teachers should incorporate indigenous ways of knowing/learning such as sharing circles as this will contextualize teaching to our lived experiences and make it culturally more relevant—this could help to break down the mistrust and the interaction problems between students and teachers and the cliquey problem we have here.*”

Another student who had experienced racist interactions between some teachers and students in the classroom shared:

“*Classroom social norms and the teaching practices of some teachers are not reflective of the values of equality*, *empowerment and respect for diversity*.”

The above findings suggest that human service programs that are delivered using traditional Eurocentric formats where students are expected to read texts, attend lectures and focus on written assignments and final grades may be failing to meet the needs of students from diverse cultures, particularly those from LIIVM backgrounds. Recent studies show that such approaches are ineffective in preparing even mainstream students for professional practice in the human services sector [38,39].

#### 4.2.3. Racism—An Ongoing Experience

Many students shared that they had chosen this college for pursuing postsecondary study because of its large multicultural and indigenous student community. They expected less discrimination based on factors like race, skin color, class and accent and this was important to them. The students’ experiences however, suggest that this was not the case and that issues of race and racism are not muted even in educational institutions colleges that have a high proportion of minority students. Students shared their experiences of racism and discrimination from some of their white teachers and peers and their general feelings about approaching authority figures like administrators and counselors. One student described one of her white instructors as:

“*She seems to be promoting inequality through: not penalizing plagiarism*, *playing favorites*, *accepting and perpetuating racist attitudes and behaviors from students and creating a hierarchical classroom structure.*”

This student also remarked that social conflicts in the classroom (involving instructors and students) were continually left unresolved by some instructors. Students appeared to be “shut down” when they tried to address these issues. The student said that this silencing had the effect of students interpreting this as a prejudicial action used to avoid confronting racism. This apparent lack of understanding from a minority of instructors left this student and some of her peers feeling (in her words):

“*Powerless*, *unable to resolve social conflicts*, *discouraged social cohesiveness and created a negative classroom atmosphere.*”

Another student shared that that poor teacher role modeling by at least one instructor was incongruent with the values of equality and social justice promoted and taught in their studies. On another level one indigenous student highlighted her experiences of covert racism from some white students. In the following excerpt she recalls her experiences of feeling alienated by her white peers:

“*Excluding you in a conversation*, *condescending stares and the ‘cliquey problem’ with distinct racial groups sticking together.*”

An indigenous student who had a learning disability had experienced ongoing race based harassment and bullying from a group of Caucasian students. When she reported her experience to an instructor she was told that she would have to tackle this on her own. Another student reported:

“*White women refuse to acknowledge there is a history and they don’t even want to talk about this history.*”

She also perceived that there was a lack of attention by the school’s leadership to address these attitudes and this led to perpetuation of these behaviors and practices by people who harbored these attitudes. The “cliquey problem” with students sticking together in distinct racial groups, is a very common occurrence that can be observed in institutions of higher learning in countries like Canada. These are strategies that students who feel oppressed may use to gain acceptance, visibility and refuge from the hegemony of the dominant group [40].

Unlike the indigenous students whose historical and political legacy made them highly conscious of and sensitive to issues of oppression and white privilege, immigrant students experienced feelings of powerlessness, guilt, self-blame, inferiority and embarrassment. The following excerpts from immigrant students capture some of these feelings:

“*So I am an immigrant—I don’t want to restructure their school system, but if you speak to anybody and they say that’s how this school system is, you feel like maybe it’s going to look like I’m from a different learning standard*, *I cannot cope with the learning standard here—or maybe it’s me*, *I wasn’t educated in this country so maybe it’s me who is having this problem.*

*I have not spoken to anybody (authority figures) but we students talk amongst ourselves and they say that’s their school system and that’s how it is.*

*I sometimes feel I’m not even supposed to complain because I don’t deserve this. This is like—they (the whites) are doing me a favor, they don’t have to do that for me. Sometimes you get that mentality because of where you come from*, *so you don’t even see the biases or anything in that system….*”

Some LIIVM students also experienced stereotyping and insecurity as evidenced from the following comment: “I tell my son—we are citizens but the rules are different for us because we are immigrants—we can be deported any time if we create trouble”. Like many others, this student did not want to be seen as a “trouble maker” by asking questions that would make her teachers, administrators or peers feel uncomfortable and create problems such as retribution from faculty and her white peers. While students expressed that the majority of their course instructors were considerate and prepared to extend time for assignments, they did not feel the same about approaching other authority figures like school administrators and advisors.

Unlike their indigenous counterparts who had access to an indigenous unit within the college, the visible minority students did not have a dominant body to raise consciousness about their experiences. They lacked a public discourse of sufficient general mainstream acceptance to fuel courage to complain or even ask questions. Their narratives suggest that remaining silent, maintaining group solidarity and sharing their experiences with group members were their ways of coping with their situation of powerlessness. Participating in this research, may have been one of their ways of making their voices heard.

##### Racism and Its Influence on Health and Well-Being

Although research on the health effects of the experiences of ongoing racism on students from LIIVM backgrounds is scarce, racism is an important social factor that can influence health and well-being [4]. Racism has consistently been identified as a cause of stress among visible minority immigrants [41] and authors have emphasized the importance of investigating the individual, institutional and cultural aspects of racism as forms of stress [42]. Studies suggest that the experience of the stress of ongoing racial oppression is articulated to psychological and physiological health pathways [43,44,45]. The more recognized pathway involves chronic mental health problems resulting from persistent psychological distress. The less acknowledged pathway involves chronic mental health problems resulting from persistent distress on the adrenal system—the body’s stress handling system [43]. The combined effect can lead to chronic physical and mental health problems like cardiovascular disease, increased hypertension, asthma, diabetes, depression, anxiety and obesity.

The students’ ongoing feelings of powerlessness, anger, guilt and shame and apprehensions about approaching fund advisors are evidence of the impact racism can have on mental health. In the following section we discuss students’ perceptions of the influence of the challenges on their health and well-being.

### 4.3. Perceived Influence of Challenges on Health and Well-Being

#### 4.3.1. Physical and Psychological Problems

Students shared that the pressures imposed by the financial assistance scheme, the demands of the course and their multiple roles took a heavy toll on their physical and mental health. They reported to experiencing a multitude of physical and mental health problems that included migraines, extreme fatigue and flashbacks of trauma from past abuse. For some the stress had triggered exacerbations of existing problems such as drinking and smoking. Others reported that they were unable to concentrate in class, had appetite disturbances such as overeating, difficulties with memory and felt constantly worn out. One participant reported that she had started experiencing panic attacks during the course of the program. The following excerpts capture the impact of stress on the students’ health and well-being:

“*I’ve never experienced stress before like what I am experiencing when I started school. I have started to taking pills for stress because I will feel it in my neck*, *I will feel my muscle—I try not to and I know its stress. Sometimes I would feel like I am drowning so I have to tell myself “stop and just relax” and don’t think about anything until your muscles start to relax because I can feel it. I never experienced those things before until I started school so I know its stress.*

*The pressure—it is affecting my memory. It’s true because before I had a very good memory but I noticed that since I started school I’m very*, *very forgetful—last term I was the type who was very attentive. But now I forget things that’s for sure. So now I try to train my memories—train my brain to remember things because I find myself forgetting things easily—very easily. It’s affecting my relationships as well …,*

*I noticed a lot of people dropped the course—when we started I think we were 40 something but now we’re less probably maybe we’re around 20 something. See lots of people dropped the course because they couldn’t take the course load …the pressure.*”

Students, particularly those whose close families were overseas missed not being able to see and interact with them on a regular basis. Some students said that they were often confronted with feelings of wanting to give up and questions like ‘is it worth it’. In the case of one immigrant student who had no family in Canada the lack of a support system had led her to the brink of homelessness, isolation and depression.

#### 4.3.2. Consulting Formal School Counseling Services—Not Part of Culture

Despite the availability of student counselors, most students in this study reported that they did not access them for a number of reasons. These included not feeling safe to divulge their life circumstances as they felt these may be used against them, not part of their culture to see counselors or doctors for personal problems, that visiting a counselor/doctor for their difficulties meant “failing”, personal inadequacy or having a mental problem, fears of being patronized, apprehensions that advisors and counselors may not understand their situation or being perceived as poor learners. Many expressed that they relied on community elders and band members, close and extended family for advice and emotional support.

#### 4.3.3. What Keeps Them Going

When asked what kept them going despite the physical and mental stress they experienced they gave several reasons. Some did not want to return to menial dead end, exploitative jobs or a life on welfare and poverty while others said that they had made huge personal sacrifices to return to study and did not want to give up. Students who experienced motivational support from family, their children, elders and band members expressed that they did not want to let down the people who had provided motivation and encouragement all through their journey through school.

Some students also expressed their belief in a superior power and the power of prayer that helped them cope with the difficulties they were experiencing. A student who had come to Canada as a refugee from a war torn country said:

“*I grew up in a home where I knew that prayers work. People pray when you have problem. I feel like relief when I’m praying and in my heart I know yes I know there’s a God. Prayer—that’s what kept us going through the war. Back home in the war that’s what we lived on—prayers.*”

Other strategies that some of these students used to cope were “using pep talks”, participating in self-care activities like meditation, yoga and exercise, and setting aside one day a week for Sabbath, family activity, or community work.

## 5. Discussion

**Figure 1:**
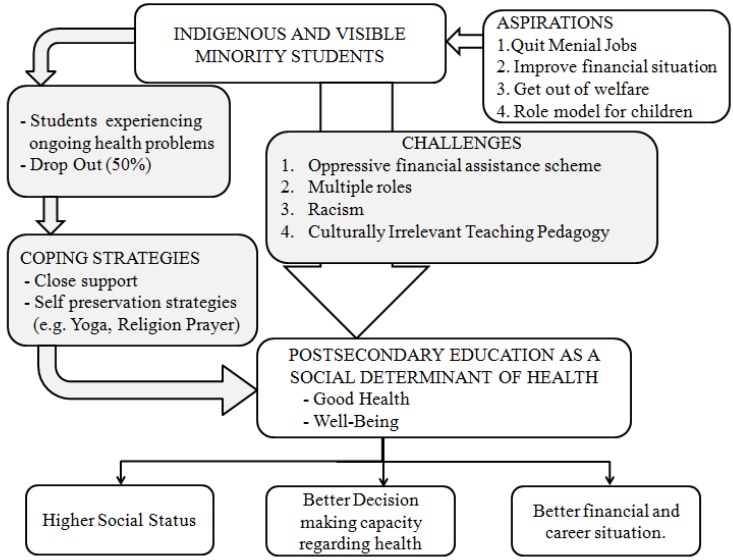
Aspirations, challenges and health perspectives of postsecondary students from LIIVM backgrounds.

This pilot study examined the career aspirations, challenges and health related perceptions of mature aged learners from low income indigenous and visible minority (LIIVM) backgrounds enrolled in a human services program in a postsecondary educational institution in Western Canada. The findings are based on a sample of 14 students from one postsecondary educational institution and are not be generalisable to LIIVM students from other postsecondary institutions in Canada. Despite this limitation the findings are significant and have important implications for policy and practice. Figure 1 is a diagrammatic representation of the key findings of the study. It illustrates the benefits of postsecondary education, the aspirations of the student participants and the challenges they faced in reaching their goals.

The three key challenges that LIIVM students faced in realizing their career aspirations and goals arose from structural and systemic factors, namely the student financial assistance scheme, institutionalized racism and teaching pedagogies based largely on Eurocentric models. From the social determinants of health perspective each of these factors may intersect in multiple ways to generate an oppressive learning environment that can adversely affect the health and well-being of students from LIIVM backgrounds. The impact of this environment most likely resulted in a high drop-out rate from the study program, and increased self-reporting of physical and mental health problems and the risk of breakdown in the case of students who participated in the study. In this context the finding that participants who had access to informal social support held on to the program suggests that access to culturally relevant support may help to buffer the adverse impact of oppressive learning environments on the health of students.

The impact of the financial assistance scheme on students’ health and the neo liberal discourses that such schemes promote has already been discussed. Similarly the impact of ongoing racism on the health of individuals has also been discussed earlier. Students’ experiences of racism (for example being excluded in conversations, condescendingly stared at, and their community’s history not being acknowledged) are in keeping with previous studies [46,47,48] and can be explained as manifestations of covert “micro aggression” by a privileged group towards those who are marginalized. The student’s experience of being told by her instructor that she would have to tackle the bullying on her own can be explained as another form of micro aggression that may be manifested by instructors in order to avoid racially charged situations conflicts with students. When instructors do not actively challenge racism, they are giving the impression that they are condoning it. They are also “projecting an attitude of color blindness which provides an excuse for mainstream students to claim they are not prejudiced” [48]. Classroom environments that do not provide the opportunity to discuss racism, prejudice and privilege can be disadvantageous to all students. As discussed earlier they can have a deleterious impact on mental health of minority students who may experience negative emotions such as lack of motivation and pride in their achievements and disengagement from study. This can act as a powerful disincentive to completing their program of study [49,50]. The impact on white students is that they will remain immersed in their privileged social groups, oblivious to ways in which their attitudes and behaviors impact on marginalized groups and maintain the structures and systems of oppression.

Teaching pedagogies based largely on Eurocentric models can also exert a significant influence on the psychological health of minority students. Eurocentric teaching approaches rely heavily on the students’ language and written presentation skills and LIIVM students who do not have good language skills may experience additional stress and fare poorly in written assessments. LIIVM often have rich cultural knowledge and work experience from their countries of origin and considerable life experience. Many have experienced war trauma, persecution, discrimination and poverty. Their lived experiences can be an invaluable asset in the human services field. Unfortunately Eurocentric teaching pedagogies provide little scope for acknowledging and incorporating in meaningful ways the rich cultural, historical and experiential knowledge that LIIVM students bring to the learning environment. This can lead to feelings of dissatisfaction and self-devaluation among these students. This can result in some students discontinuing enrollment [17,51] and sliding into un/underemployment, poverty and poor health. Finally Eurocentric teaching pedagogies contribute minimally to the development of critical consciousness in students as issues of race, oppression and privilege are rarely confronted or discussed in class situations. This maintains the positions of oppression, power and privilege among students that will be carried over to their work situations.

### 5.1. Practice and Policy Implications

Creating learning environments for LIIVM students that will help to maintain their health and well-being during the course of their study calls for changes at different levels and the collaborative input of stakeholders from Federal, Provincial and postsecondary educational sectors. In the following section we discuss some of the practice and policy implications of the findings.

### 5.2. Making Financial Assistance Schemes Work for Low Income Minority Student

The State-sponsored financial assistance scheme for students from low income backgrounds is an ideology-based neo liberal policy that aims to move able bodied people on welfare (or those at risk of moving into welfare) into employment so that they do not depend on the state for a livelihood—a code for “so they do not take advantage of the mainstream supporters of the government—the taxpayers”. From this perspective people from LIIVM backgrounds are a group that is at risk of becoming welfare dependent. The public face of this neo liberal policy is to help poor and needy students to gain postsecondary education and support the discourse that education is a determinant of health. Ironically the scheme became counter-productive as it contributed to constructing oppressive conditions for the students and put them at risk of having health issues by virtue of being clients of a “helping” scheme. If this scheme is to serve its intended purpose, it must factor in flexibility as opposed to the expectation that students benefit from “supplementary” income (expecting students to work, use their savings or seek family contributions to help with their educational costs). The multiple realities that these students face on a regular basis must be considered, in addition to the number of courses required each term in order to maintain eligibility. Provision of affordable child care services specifically for students will minimize the chances of children being left alone at home without supervision and the risk of interference from child protection services. Adopting a “one size fits all” approach for the delivery of this scheme and expecting that all students from low income marginalized backgrounds experience the same hardships, and should therefore be subjected to the same criteria for the same amount of financial assistance reflects the false premise that social discrimination is non-existent, that the experiences of all students eligible for financial assistance are equal, and therefore identical, regardless of race or ethnicity [28,52,53]. This trivializes the realities of minority students and ignores the negative impacts of ongoing racism on health and well-being.

Financial assistance schemes must also have built in processes to establish transparency within their conditions and regulations, while allowing for anonymity for those who enquire about various options without fear of loss of benefits or other negative consequences. A system which relies on ignorance cannot also claim to assist others in the dissemination and acquisition of knowledge. Students must have easy access to resources that can help them to understand their rights and obligations with respect to financial assistance. Fund administrators and advisors have a crucial role to play in ensuring that LIIVM students accessing these schemes are not disadvantaged because of their race and class. To this end educational institutions must ensure that people hired for these positions are knowledgeable about the scheme, understand the realities of class and race discrimination and are able to interact with students in anti-oppressive ways. This includes being aware of their social locations of power and privilege and how these can distance them from students who need their advice and support. Without these significant changes, both at the levels of policy and its implementation, financial assistance schemes will not only fail as demonstrated by the testimonies of the students in this study but will put students at risk for developing physical and mental health issues.

### 5.3. Addressing Racism

While the impact of ongoing racism on health has been discussed earlier, the pathways through which it affects health include biased information about groups and communities that leads to stereotyping, prejudice, discrimination and oppression [43]. The experiences of the students in this study highlight that they experienced these overtly and covertly both in their classrooms and at the institution level. Recent studies suggest that a shift to a critical multicultural teaching approach in human services programs has the potential to minimize the impact of racism in classrooms. As discussed by Nyland [54], this approach can create pedagogical conditions in which students can interrogate conditions of “otherness”, will make visible the historical and social construction of whiteness and privilege and will give white students the opportunity to critically reflect and deconstruct what being “white” means to them”. It can help them move past their feelings of guilt and denial to reformulate their identities in ways that challenge dominant interests. This perspective is not intended to shame middle class majority students—but rather to assist them in better understanding how they may be contributing to oppression and systems that breed inequality and social injustice. In the case of the marginalized students a critical multicultural approach can also be liberating and empowering. However their experiences must also be documented and in the process they should not be rendered invisible or their personal stories used mainly to teach white students about racism and white privilege. Research into how this approach can be successfully incorporated into existing Human Services Program is still in the early stages of development. At the institutional level—the impact of racism on the health and well-being of LIIVM students can be counteracted by increasing the representation of minority staff at faculty and administration levels. These staff must be vested with enough power to take an active role in ensuring that the voices of marginalized students are heard and that policies and practices that are discriminatory are investigated and changed.

### 5.4. Introducing Culturally Relevant Teaching and Learning Pedagogy

Students from diverse backgrounds like indigenous, African and Asian Americans may learn better in collective and cooperative teaching and learning environments where life experiences and knowledge can be shared among learners, rather than individualist and competitive environments favored by the mainstream culture. Instructors must therefore strive to incorporate a blend of teaching and learning approaches that can benefit all students. In such environments indigenous and visible minority students will feel motivated and acknowledged for their contributions to the learning experience while mainstream students will be introduced to news ways of learning that may be more fulfilling and enriching.

### 5.5. Other Structural Supports to Empower Marginalized Students

The groundwork for this can be secured by helping indigenous and visible minority students to build and expand upon their existing networks, such as ethnic community organizations and leaders, churches or other places of worship, families, and welfare organizations that provide practical forms of help. Student services can also help to establish a strong student mentoring support system that can take an active role in helping marginalized students to develop resources and community networks. They can also introduce student peer support groups where minority students can voice their concerns without fear and peer leaders can ensure that these are conveyed to decision makers responsible for making changes in existing systems that perpetuate discrimination. Since students from visible minority backgrounds may not access mainstream student counseling services, the educational institution must be proactive in ensuring that ethnic counselors are available to students when they need them.

## 6. Conclusions

Education is a social determinant of health. Although the findings of this study are based on a small sample of students from one community college in Canada and therefore cannot be generalized, they highlight the importance of developing a more inclusive postsecondary education system that is truly committed to the principles of anti-discrimination and antiracism. This can go a long way towards helping LIIVM students and other low income students to achieve their educational goals while maintaining their health and well-being.
